# Interference between rheumatoid arthritis and autoimmune thyroid diseases: A bidirectional Mendelian randomization

**DOI:** 10.1097/MD.0000000000042188

**Published:** 2025-04-18

**Authors:** Yuman Yin, Min Liao, Yunfeng Yu, Gang Hu, Xinyu Yang, Chenlu Guo, Rong Yu

**Affiliations:** a School of Traditional Chinese Medicine, Hunan University of Chinese Medicine, Changsha, Hunan, China; b People’s Hospital of Ningxiang City, Changsha, Hunan, China; c The First Hospital of Hunan University of Chinese Medicine, Changsha, Hunan Province, China.

**Keywords:** autoimmune thyroid disease, autoimmune thyroiditis, Graves’ disease, Mendelian randomization, rheumatoid arthritis

## Abstract

The relationship between rheumatoid arthritis (RA) and autoimmune thyroid disease (AITD) remains controversial. This study aimed to analyze the causal relationship between RA and AITD by bidirectional Mendelian randomization (MR). Single nucleotide polymorphisms for RA, autoimmune thyroiditis (AIT), and Graves’ disease (GD) were obtained from the FinnGen database. Inverse variance weighted was chose to assess the causal relationships between RA and AITD. MR-Egger, Cochran *Q*, and sensitivity analyses were used to assess horizontal pleiotropy, heterogeneity, and robustness of the MR results, respectively. MR analysis showed that RA was associated with increased risk of AIT (odds ratio [OR] 1.552, 95% confidence interval [CI] 1.266 to 1.903, *P* < .001) and GD (OR 1.177, 95% CI 1.076–1.286, *P* < .001). Furthermore, GD was associated with an increased risk of RA (OR 1.030, 95% CI 1.007–1.053, *P* = .010), whereas AIT was not associated with the risk of RA (OR 0.994, 95% CI 0.979–1.009, *P* = .420). These results lacked horizontal pleiotropy and heterogeneity, and were considered robust. This MR analysis indicates that RA may act as a potential risk factor for AIT and GD, while GD also appears to pose a potential risk for RA. We recommend proactive screening for AIT and GD in patients diagnosed with RA, as well as screening for RA in patients with GD. Early and active screening, coupled with appropriate care and management, will help mitigate the risk of complications and improve patient outcomes.

## 1. Introduction

Rheumatoid arthritis (RA) is a chronic inflammatory arthritis characterized by symmetric synovial inflammation, progressive bone and cartilage erosion, and autoantibody formation.^[1,2]^ The prevalence of RA has been reported to be approximately 0.72% in the American population,^[3]^ and is 2 to 3 times more common in females than in males.^[4]^ Joint pain, stiffness, swelling, deformity, and dysfunction are the main clinical manifestations of RA, and they seriously affect the physical and mental health of patients as well as their quality of life.^[5]^ Untreated active RA has been reported to significantly increase the risk of disability and cardiovascular complications in patients.^[6,7]^ The pathogenesis of RA is not clearly understood, but it is generally thought to be related to the combined effects of genetic and environmental factors.^[8]^ Family history of RA, female sex, smoking, and obesity are considered to be the major risk factors for RA.^[9]^ As studies progressed, a close relationship between autoimmune thyroid disease (AITD) and RA has been increasingly recognized.^[10]^

AITD is an autoimmune disease characterized by the breakdown of the immune system’s tolerance to thyroid antigens,^[11]^ and includes 2 main types, Graves’ disease (GD) and autoimmune thyroiditis (AIT).^[12]^ GD is an autoimmune disease caused by thyroid receptor antibodies targeting TSH receptors, and is characterized by hyperthyroidism and goiter.^[13,14]^ AIT is an autoimmune disease caused by thyroid peroxidase antibodies (TPOAb) and thyroglobulin antibodies (TGAb) targeting thyroid tissue, which is the leading cause of hypothyroidism.^[15]^ Although some studies have suggested that AITD and RA, both autoimmune diseases, may increase each other’s risk,^[16,17]^ there is no direct evidence supporting a causal relationship between RA and AIT or RA and GD.

Mendelian randomization (MR), an epidemiological approach, is frequently employed to investigate the causal relationship between exposure and outcome.^[18,19]^ Since MR uses genetic variation as an instrumental variable, it is effective in avoiding confounding variables caused by changes in related traits.^[20,21]^ This study aims to use bidirectional MR to analyze the causal relationship between RA and AIT, as well as RA and GD, from the perspective of genetic prediction.

## 2. Materials and methods

### 2.1. Study design

**Figure 1.**
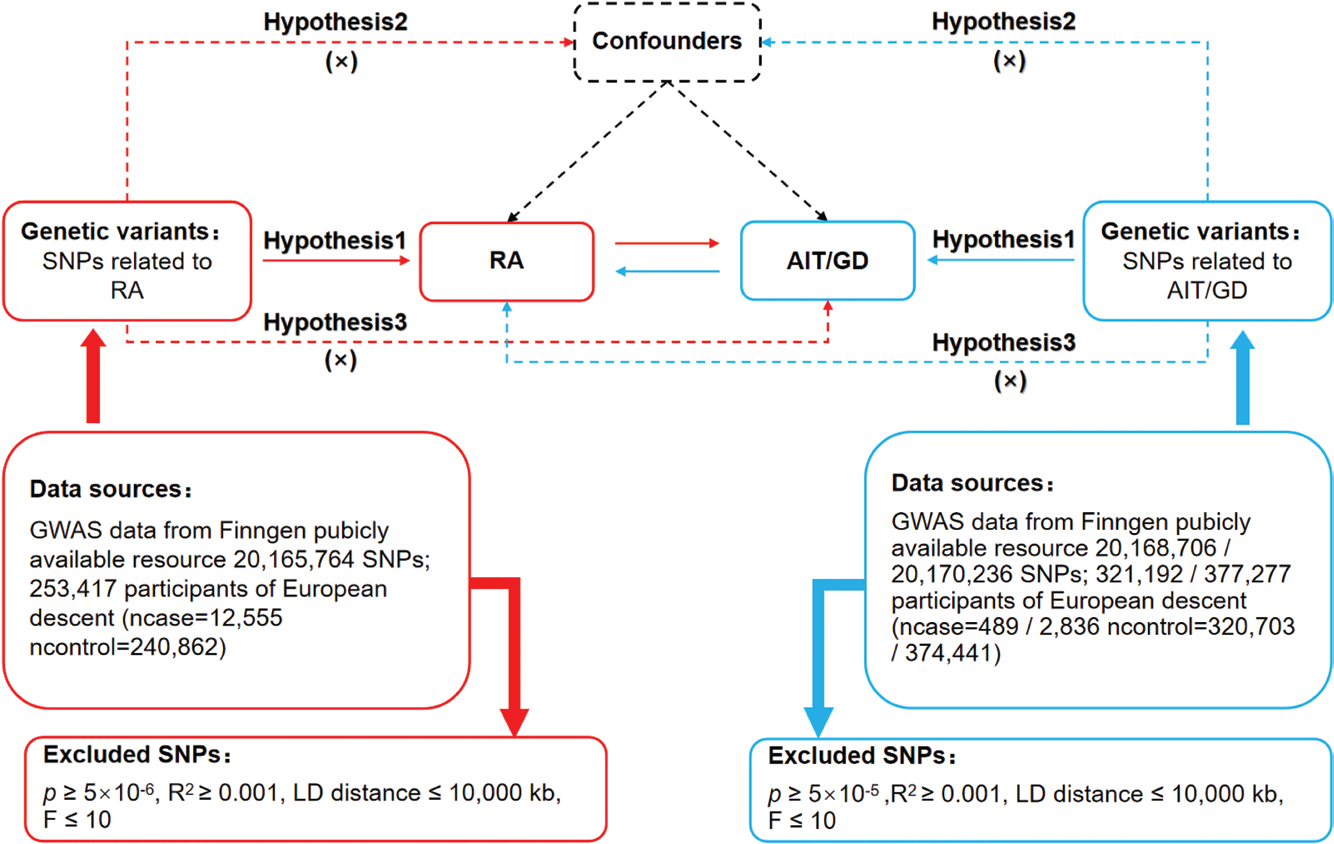
Bidirectional MR design for causal analysis of RA and AITD. AIT = autoimmune thyroiditis, AITD = autoimmune thyroid disease, GD = Graves’ disease, RA = rheumatoid arthritis.

MR relied on 3 basic hypotheses^[20]^: (1) Association hypothesis (Hypothesis 1): Single nucleotide polymorphisms (SNPs) were closely related to exposure factors. (2) Independence hypothesis (Hypothesis 2): SNPs were independent from confounding variables. (3) Exclusivity hypothesis (Hypothesis 3): SNPs were unable to act on outcome variables through pathways other than exposure factors. The bidirectional MR design for RA and AITD is illustrated in Figure 1.

### 2.2. Data sources

The data for RA, AIT, and GD were obtained from FinnGen (www.finngen.fi/fi), as presented in Table 1. The data for RA contained 253,417 Europeans, dataset number: finngen_R9_M13_RHEUMA. The data for AIT contained 321,192 Europeans, dataset number: finngen_R9_E4_THYROIDITAUTOIM. The GD data contained 377,277 Europeans, dataset number: finngen_R9_E4_GRAVES_STRICT. Ethical approval was not required for this study as all data were publicly available.

**Table 1:** Details of the GWAS studies included in the Mendelian randomization.

Year	Trait	GWAS ID	Population	Sample size	Web source
2023	RA	finngen_R9_M13_RHEUMA	European	253,417	www.finngen.fi/fi
2023	AIT	finngen_R9_E4_THYROIDITAUTOIM	European	321,192	www.finngen.fi/fi
2023	GD	finngen_R9_E4_GRAVES_STRICT	European	377,277	www.finngen.fi/fi

AIT = autoimmune thyroiditis, GD = Graves’ disease, GWAS = genome-wide association study, RA = rheumatoid arthritis.

### 2.3. Selection of genetic instrumental variables

First, SNPs closely associated with RA were searched for in the dataset according to *P* < 5 × 10^‐6^, as well as *P* < 5 × 10^‐5^ for SNPs closely associated with AIT and GD to fulfill Hypothesis 1. Second, independent SNPs were screened according to *R*^2^ < 0.001 and kb = 10,000 to avoid bias due to linkage disequilibrium. Third, the *F* value of each SNP was calculated and SNPs with *F* ≤ 10 were excluded, where *F* was computed as: *F* = [*R*^*2*^/(1 ‐ *R*^*2*^)] * [(*N* ‐ *K* ‐ 1)/*K*]. Here, *R*^2^ denoted the cumulative explained variance of the selected IVs on exposure, N represented the sample size of the GWAS, and K was the number of paired samples. Fourth, the PhenoScanner database was reviewed, and SNPs potentially related to confounding factors were removed to fulfill Hypothesis 2. Fifth, mismatched SNPs were removed based on the effect of allele frequency when adjusting allele orientation. Finally, aberrant SNPs were removed by MR-Pleiotropy RESidual Sum and Outlier method.

### 2.4. Data analysis

The study followed the STROBE-MR guidelines,^[22]^ and employed bidirectional MR to assess the causal relationship between RA and AITD. MR analyses and data visualization were performed using the R 4.3.1, with inverse variance weighted (IVW), MR-Egger, and weighted median as the assessment of causal relationships. Among them, IVW is the main analytical method,^[23]^ which could achieve unbiased causal estimation without horizontal pleiotropy and is the most valuable reference. MR-Egger and weighted median were used as complementary methods to MR analysis, with the former providing valid causal estimates in some cases where pleiotropy existed and the latter being less sensitive to outliers and measurement error. Subsequently, horizontal pleiotropy was assessed using MR-Egger intercept, with *P* ≥ .05 suggesting the absence of horizontal pleiotropy to fulfill Hypothesis 3. Next, heterogeneity was assessed using Cochran *Q*, with *P* ≥ .05 suggesting the absence of heterogeneity. Finally, a leave-one-out sensitivity analysis was utilized to assess the robustness of the MR results, and no significant change in the combined effect sizes suggested that the results were robust.

## 3. Results

### 3.1. Genetic instrumental variables

We included 62 SNPs to analyze the causal effects of RA on AIT, 56 SNPs to analyze the causal effects of RA on GD, 51 SNPs to analyze the causal effects of AIT on RA, and 98 SNPs to analyze the causal effects of GD on RA, as shown in Tables S1–S4, Supplemental Digital Content, https://links.lww.com/MD/O684.

### 3.2. Bidirectional MR analysis

#### 3.2.1. Causal effect between RA on AIT

**Figure 2.**
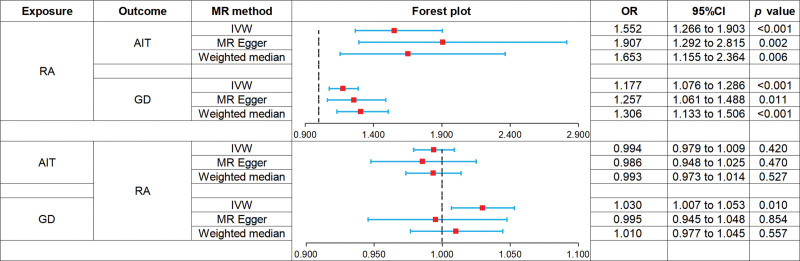
Forest plot of MR analysis on the causal relationship between RA and AITD. AIT = autoimmune thyroiditis, AITD = autoimmune thyroid disease, GD = Graves’ disease, RA = rheumatoid arthritis .

**Figure 3.**
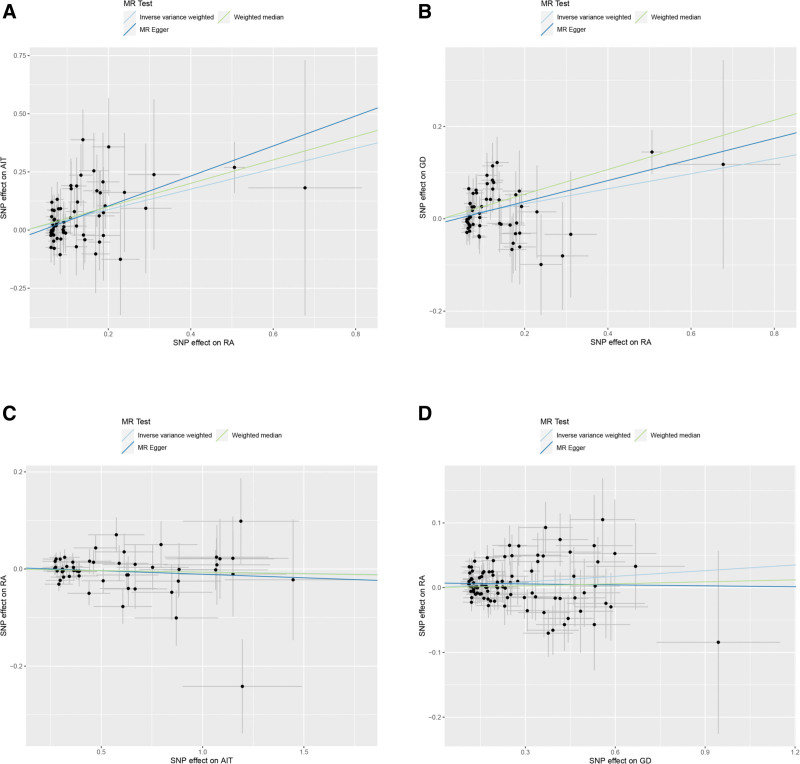
Scatter plot of MR analysis on the causal relationship between RA and AITD. (A) RA on AIT; (B) RA on GD; (C) AIT on RA; (D) GD on RA. AIT = autoimmune thyroiditis, AITD = autoimmune thyroid disease, GD = Graves’ disease, RA = rheumatoid arthritis.

All 3 analyses indicated that RA was associated with an increased risk of AIT: IVW (odds ratio [OR] 1.552, 95% confidence interval [CI] 1.266 to 1.903, *P* < .001), MR-Egger (OR 1.907, 95% CI 1.292–2.815, *P* = .002), weighted median (OR 1.653, 95% CI 1.155–2.364, *P* = .006). The forest plot is shown in Figure 2 and the scatter plot is presented in Figure 3(A). MR-Egger analysis showed no horizontal pleiotropy (*P* = .229), as shown in Table S5, Supplemental Digital Content, https://links.lww.com/MD/O685.

#### 3.2.2. Causal effect between RA on GD

All 3 analyses showed that RA was associated with an increased risk of GD: IVW (OR 1.177, 95% CI 1.076–1.286, *P* < .001), MR-Egger (OR 1.257, 95% CI 1.061–1.488, *P* = .011), and weighted median (OR 1.306, 95% CI 1.133–1.506, *P* < .001). The forest plot is shown in Figure 2 and the scatter plot is shown in Figure 3(B). MR-Egger analysis showed no horizontal pleiotropy (*P* = .373), as shown in Table S5, Supplemental Digital Content, https://links.lww.com/MD/O685.

#### 3.2.3. Causal effect between AIT on RA

All 3 analytical methods revealed that AIT was not associated with the risk of RA: IVW (OR 0.994, 95% CI 0.979–1.009, *P* = .420), MR-Egger (OR 0.986, 95% CI 0.948–1.025, *P* = .470), weighted median (OR 0.993, 95% CI 0.973–1.014, *P* = .527). The forest plot is presented in Figure 2 and the scatter plot is illustrated in Figure 3(C). MR-Egger analysis showed no horizontal pleiotropy (*P* = .652), as shown in Table S5, Supplemental Digital Content, https://links.lww.com/MD/O685.

#### 3.2.4. Causal effect of GD on RA

IVW indicated that GD was associated with an increased risk of RA (OR 1.030, 95% CI 1.007–1.053, *P* = .010), whereas MR-Egger (OR 0.995, 95% CI 0.945–1.048, *P* = .854) and weighted median (OR 1.010, 95% CI 0.977–1.045, *P* = .557) did not observe this effect. The forest plot is shown in Figure 2 and the scatter plot is depicted in Figure 3(D). MR-Egger analysis revealed no evidence of horizontal pleiotropy (*P* = .152), as shown in Table S5, Supplemental Digital Content, https://links.lww.com/MD/O685.

### 3.3. Heterogeneity and sensitivity analyses

**Figure 4.**
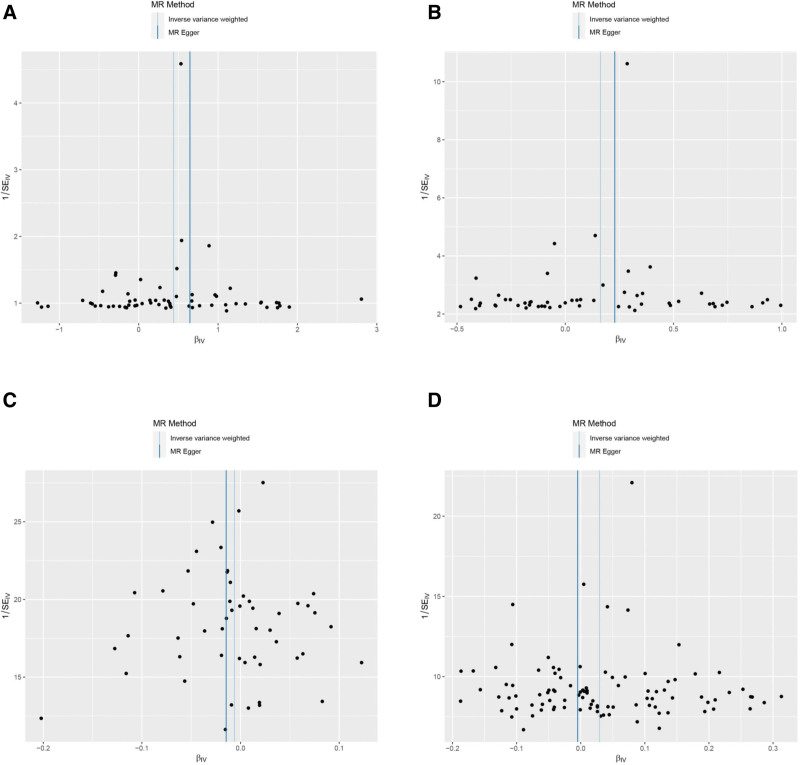
Funnel plot of heterogeneity analysis on RA and AITD. (A) RA on AIT; (B) RA on GD; (C) AIT on RA; (D) GD on RA. AIT = autoimmune thyroiditis, AITD = autoimmune thyroid disease, GD = Graves’ disease, RA = rheumatoid arthritis.

**Figure 5.**
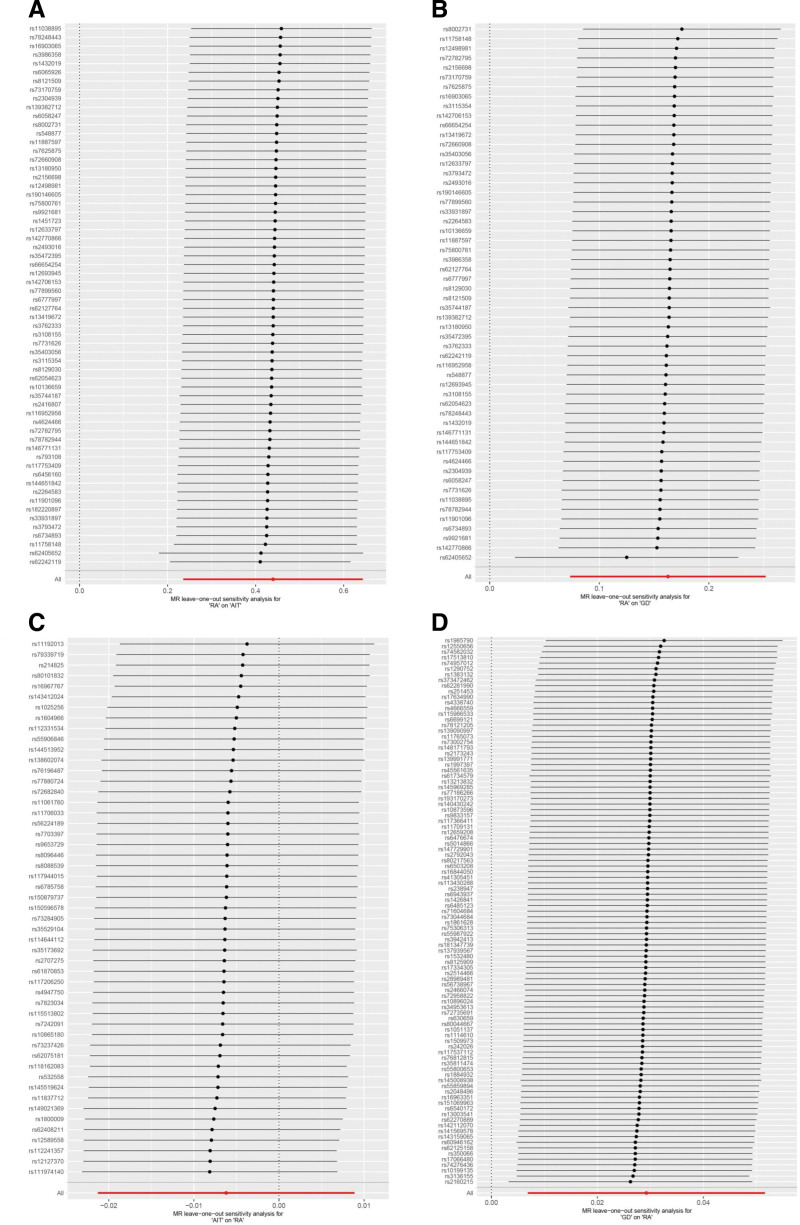
Leave-one-out sensitivity analysis on RA and AITD. (A) RA on AIT; (B) RA on GD; (C) AIT on RA; (D) GD on RA. AIT = autoimmune thyroiditis, AITD = autoimmune thyroid disease, GD = Graves’ disease, RA = rheumatoid arthritis.

Cochran *Q* indicated no heterogeneity in the above MR analysis results (*P* ≥ .05), as shown in Figure 4 and Table S6, Supplemental Digital Content, https://links.lww.com/MD/O686. Leave-one-out sensitivity analysis demonstrated that these results were robust, as shown in Figure 5.

## 4. Discussion

### 4.1. Research significance and findings

To our knowledge, this is the first MR analysis to evaluate the causal relationship between RA and AITD. Our MR analysis demonstrated that RA was associated with an increased risk of AIT and GD in Europeans; however, GD but not AIT was associated with an increased risk of RA in Europeans. These findings exhibit no heterogeneity or horizontal pleiotropy, and sensitivity analyses suggested that the MR results were robust.

### 4.2. Effect of RA on the risk of AITD

Current studies indicate that RA is involved in the onset and progression of AITD or AIT, but there is no evidence to support the relationship between RA and the risk of GD. First, RA has been reported to be associated with an increased incidence of AITD. Przygodzka et al^[24]^ followed up 100 female patients with RA who were treated at the Polish Institute of Rheumatology from 2001 to 2005, founding that the prevalence of AITD in patients with RA was significantly higher than that in patients without RA (16% vs 9%). A case-control and cohort study in Sweden showed that the prevalence of AITD was increased by approximately 50% (OR 1.5, 95% CI 1.4–1.7) in patients with RA compared to a randomized population.^[25]^ Surprisingly, another case-control study in Iran reported that RA increased the risk of AITD in Iranians by 177% (OR 2.77, 95% CI 1.62–4.73),^[16]^ a value that is much higher than the data reported by clinical studies in Sweden and Poland. These findings point to the fact that patients with RA possess a higher risk of AITD and that this risk may be more pronounced in Iranians.

Furthermore, there is also some literature pointing to the fact that RA may be associated with a higher risk of AIT. A cross-sectional observational study in Spain showed that patients with RA had a significantly increased probability of concurrent autoimmune diseases, with the incidence of HT being 26.7%.^[26]^ A study involving 131 French individuals revealed that the prevalence of AIT in patients with Sjögren’s syndrome, RA, and other rheumatic diseases was 100%, 16.2%, and 11.7%, respectively, indicating that AIT is a common complication of rheumatic diseases.^[27]^ Additionally, RA has been found to be associated with increased positive for the TPOAb and TGAb, AIT-specific antibodies. A cohort study conducted in Colombia revealed that the positive rates of TPOAb and TGAb in patients with RA were 37.8% and 20.8%, respectively,^[28]^ which were significantly higher than in the general population. A meta-analysis that included 1021 cases of RA and 1500 healthy controls suggested that the positive rates of TGAb and TPOAb in patients with RA were increased by 217% (OR 3.17, 95% CI 2.24–4.49) and 133% (OR 2.33, 95% CI 1.24–4.39), respectively, compared to healthy individuals.^[29]^ These findings point to RA as a potential risk factor for AIT.

However, there is currently no literature reporting the relationship between RA and the risk of GD, which will be a focal point for future studies.

### 4.3. Effect of AITD on the risk of RA

Notably, this MR analysis found that AIT was not associated with the risk of RA, which is quite different from the current report. Fallahi et al conducted a study involving 3069 patients with AIT, revealing that 2.4% of patients with AIT had RA.^[30]^ A study in the United Kingdom involving 495 patients with HT, indicated that RA was the most common coexisting autoimmune disease in patients with HT, with a prevalence of 4.24% in HT.^[17]^ Although these studies imply that AIT is associated with an increased risk of RA, they did not compare the prevalence of RA between patients with AIT and those without AIT under conditions that controlled for variables. There is currently insufficient evidence to support AIT as a risk factor for RA.

Furthermore, our MR also revealed that GD increases the risk of RA, which is consistent with existing clinical findings. Boelaert et al^[17]^ found that the prevalence of RA in patients with GD in the UK was 3.15%, compared with a previously reported the prevalence of only 1% in the general British population.^[31]^ A prospective study in Italy demonstrated that the prevalence of RA in patients with GD was approximately 0.9%, which was significantly higher than the 0.08% in healthy controls.^[32]^ These studies support GD as a potential risk factor for RA. However, there are currently no clinical studies reporting the impact of RA on the risk of GD. Notably, a previous MR analysis showed that among Asians, RA increased the risk of GD by 39% (OR 1.39, 95% CI 1.10–1.75), and GD increased the risk of RA by 30% (OR 1.30, 95% CI 0.94–1.8).^[33]^ However, our MR analysis indicated that these additional risks were only 17.7% (OR 1.177, 95% CI 1.076–1.286) and 3% (OR 1.03, 95% CI 1.007–1.053) in Europeans, implying that the effects of RA and GD on each other may be more significant in Asians. In conclusion, GD and RA may be potential risk factors for each other.

### 4.4. Limitations and perspectives

This MR analysis has several limitations. First, the dataset is from Europeans only, so the results mainly reflect the causal relationship between RA and AITD in European descent populations and may not apply to other races. Second, it shows AIT has no association with RA risk, contrary to clinical reports, and further research is needed to explain this inconsistency. Third, although it suggests GD increases RA risk, there is a lack of clinical evidence. In light of these limitations, future studies are expected to make improvements. Large-sample clinical studies should be carried out to explore the impact of AIT and GD on RA risk and provide clinical evidence for their causal relationship. Additionally, global human genome research should be promoted to offer more comprehensive data for MR analysis across different races.

## 5. Conclusion

This MR analysis suggests that RA is a potential risk factor for AIT and GD, and GD is also a potential risk factor for RA, while AIT is not associated with the risk of RA. We recommend that patients with RA be actively screened for AIT and GD, and likewise, patients with GD be actively screened for RA. More studies are needed in the future to continue to explore the interrelationship between RA and AITD.

## Author contributions

**Conceptualization:** Yuman Yin, Rong Yu.

**Data curation:** Yunfeng Yu.

**Formal analysis:** Gang Hu, Xinyu Yang.

**Methodology:** Min Liao, Yunfeng Yu.

**Supervision:** Yuman Yin, Rong Yu.

**Writing – original draft:** Yuman Yin, Yunfeng Yu, Gang Hu, Xinyu Yang.

**Writing – review & editing:** Min Liao, Chenlu Guo, Rong Yu.

## Supplementary Material


