# Supplementary material for: Interference between rheumatoid arthritis and autoimmune thyroid diseases: A bidirectional Mendelian randomization
Source: Medicine (Baltimore). 2025 Apr 18;104(16):e42188. doi: 10.1097/MD.0000000000042188 (PMC12014076; doi:10.1097/MD.0000000000042188)
Supplement: Supplementary file 1 [file medi-104-e42188-s001.pdf]

Supplementary Table S1 Information of identified SNPs in exposure (RA) and outcome (AIT)

				Exposure (RA)			Outcome (AIT)				
	SNP	EA	OA	β	SE	*p* value	Case	Control	β	SE	*p* value
1	rs10136659	A	G	0.310785	0.0629942	8.07E-07	489	320,703	0.238357	0.32326	0.460908
2	rs11038895	T	C	0.0831163	0.0166742	6.21E-07	489	320,703	-0.105917	0.082771	0.200671
3	rs116952958	A	G	0.187105	0.0402503	3.34E-06	489	320,703	0.207132	0.21146	0.327316
4	rs11758148	C	A	0.180602	0.019131	3.72E-21	489	320,703	0.160087	0.0970953	0.0991973
5	rs117753409	A	C	0.109919	0.022981	1.73E-06	489	320,703	0.177604	0.117331	0.130102
6	rs11887597	C	T	-0.0607533	0.0132693	4.68E-06	489	320,703	0.0230227	0.0642776	0.720211
7	rs11901096	A	G	0.109304	0.0234208	3.06E-06	489	320,703	0.19115	0.117302	0.103196
8	rs12498981	G	A	0.0912604	0.0175126	1.88E-07	489	320,703	-0.00410887	0.0872236	0.962428
9	rs12633797	G	A	-0.0662484	0.0138164	1.63E-06	489	320,703	-0.00286365	0.0666688	0.965739
10	rs12693945	G	A	-0.0733765	0.0155595	2.41E-06	489	320,703	-0.0187217	0.0749703	0.802802
11	rs13180950	C	T	0.0912518	0.0179306	3.60E-07	489	320,703	-0.0103462	0.0887563	0.907202
12	rs13419672	T	C	0.188114	0.0390007	1.41E-06	489	320,703	0.0745541	0.195003	0.702221
13	rs139382712	T	C	0.229431	0.0464976	8.05E-07	489	320,703	-0.125644	0.239822	0.600344
14	rs142706153	C	T	-0.290994	0.0623674	3.07E-06	489	320,703	-0.0935893	0.278437	0.736777
15	rs142770866	A	G	-0.122839	0.0250456	9.36E-07	489	320,703	-0.0173852	0.117781	0.882654
16	rs1432019	T	C	0.0688011	0.0147241	2.97E-06	489	320,703	-0.0785907	0.0721856	0.276272
17	rs144651842	A	G	0.123185	0.0240429	3.00E-07	489	320,703	0.189498	0.122591	0.122159
18	rs1451723	T	C	0.0623743	0.013285	2.66E-06	489	320,703	-0.00148044	0.0644411	0.981671
19	rs146771131	C	T	0.0684859	0.0141539	1.31E-06	489	320,703	0.0840215	0.0690502	0.223673
20	rs16903065	A	C	-0.143169	0.0213703	2.09E-11	489	320,703	0.0417601	0.100878	0.678899
21	rs182220897	G	C	0.201008	0.0401944	5.71E-07	489	320,703	0.357595	0.209267	0.0874883
22	rs190146605	C	G	-0.188073	0.0407833	4.00E-06	489	320,703	0.0233665	0.19386	0.904061
23	rs2156698	A	G	-0.0609414	0.0133265	4.81E-06	489	320,703	0.0104398	0.0648696	0.872144
24	rs2264583	C	T	0.164927	0.0359557	4.50E-06	489	320,703	0.254655	0.162713	0.117569
25	rs2304939	G	A	-0.122462	0.0265554	4.00E-06	489	320,703	0.0716519	0.123786	0.5627
26	rs2416807	G	A	-0.0625331	0.0132679	2.44E-06	489	320,703	-0.057587	0.0645291	0.372168
27	rs2493016	T	C	0.092075	0.0185394	6.82E-07	489	320,703	0.0140381	0.0917361	0.878377
28	rs3108155	G	C	-0.0782243	0.0148348	1.34E-07	489	320,703	-0.0371907	0.0711007	0.600925
29	rs3115354	A	G	0.239276	0.0509824	2.69E-06	489	320,703	0.162027	0.256251	0.527191
30	rs33931897	C	G	0.0629934	0.0137412	4.56E-06	489	320,703	0.119527	0.0668784	0.0739009
31	rs35403056	A	G	-0.0684644	0.0148221	3.85E-06	489	320,703	-0.043428	0.0716078	0.544203
32	rs35472395	G	A	-0.677263	0.13789	9.03E-07	489	320,703	-0.18161	0.548928	0.740761
33	rs35744187	A	G	0.192444	0.0192957	1.99E-23	489	320,703	0.104053	0.0992455	0.294435
34	rs3762333	C	A	0.0740304	0.0158928	3.19E-06	489	320,703	0.030066	0.0787431	0.702592
35	rs3793472	A	G	-0.0760219	0.0153309	7.10E-07	489	320,703	-0.13204	0.0753808	0.0798344

36	rs3986358	C	T	-0.061116	0.0133152	4.43E-06	489	320,703	0.0748157	0.06496	0.249436
37	rs4624466	T	C	0.0841543	0.0173561	1.24E-06	489	320,703	0.092232	0.0862784	0.285068
38	rs548877	G	A	0.0964878	0.0170291	1.46E-08	489	320,703	-0.0130391	0.0846644	0.877603
39	rs6058247	T	C	-0.0759427	0.0164082	3.69E-06	489	320,703	0.0363035	0.0789054	0.645452
40	rs6065926	G	A	0.08477	0.0149909	1.56E-08	489	320,703	-0.0385195	0.0720076	0.592693
41	rs62054623	G	A	-0.117749	0.0218803	7.39E-08	489	320,703	-0.0793199	0.104443	0.447578
42	rs62127764	G	C	-0.064906	0.0134939	1.51E-06	489	320,703	-0.025493	0.0653117	0.696295
43	rs62242119	T	A	0.138435	0.0298171	3.44E-06	489	320,703	0.388414	0.130559	0.00292975
44	rs62405652	C	A	-0.505959	0.025491	1.13E-87	489	320,703	-0.269505	0.11029	0.0145415
45	rs6456160	C	T	-0.0792677	0.0132719	2.34E-09	489	320,703	-0.0914341	0.0648334	0.158454
46	rs66654254	A	G	-0.0680291	0.0134789	4.49E-07	489	320,703	-0.014548	0.0653613	0.823863
47	rs6734893	G	A	0.133673	0.0284428	2.61E-06	489	320,703	0.236113	0.133454	0.0768529
48	rs6777997	G	C	0.0919616	0.018047	3.48E-07	489	320,703	0.0344878	0.0893828	0.699613
49	rs72660908	G	C	-0.0640081	0.0139839	4.71E-06	489	320,703	0.0154014	0.0673021	0.818993
50	rs72782795	G	A	-0.172334	0.0330331	1.82E-07	489	320,703	-0.168901	0.156255	0.27973
51	rs73170759	T	A	0.16943	0.0341321	6.91E-07	489	320,703	-0.102641	0.1693	0.544337
52	rs75800761	C	T	0.1397	0.0302	3.73E-06	489	320,703	-0.0213774	0.150083	0.886735
53	rs7625875	A	C	0.0676451	0.0146608	3.95E-06	489	320,703	-0.0216003	0.0708494	0.760461
54	rs7731626	A	G	-0.108014	0.0149385	4.81E-13	489	320,703	-0.0519752	0.0711507	0.465088
55	rs77899560	G	A	0.177905	0.037422	1.99E-06	489	320,703	0.0609324	0.192096	0.751093
56	rs78248443	T	C	-0.178636	0.0262275	9.69E-12	489	320,703	0.0515256	0.122855	0.674923
57	rs78782944	T	C	0.125311	0.0219434	1.13E-08	489	320,703	0.120502	0.11151	0.279858
58	rs793108	T	C	0.0641362	0.0133925	1.68E-06	489	320,703	0.0862141	0.064911	0.184116
59	rs8002731	C	A	-0.0902776	0.0138528	7.18E-11	489	320,703	-0.00218038	0.0666834	0.973916
60	rs8121509	C	T	0.0674888	0.0133207	4.05E-07	489	320,703	-0.0476361	0.0648259	0.462443
61	rs8129030	A	T	0.0695223	0.0139712	6.49E-07	489	320,703	0.0465092	0.0673083	0.489574
62	rs9921681	T	C	0.0654854	0.0140387	3.09E-06	489	320,703	-0.00308098	0.0682014	0.963968

SNP, single nucleotide polymorphism; EA, effect allele; OA, other allele; SE, standard error; RA, rheumatoid arthritis; AIT, autoimmune thyroiditis.

Supplementary Table S2 Information of identified SNPs in exposure (RA) and outcome (GD)

				Exposure (RA)			Outcome (GD)				
	SNP	EA	OA	β	SE	*p* value	Case	Control	β	SE	*p* value
1	rs10136659	A	G	0.310785	0.0629942	8.07E-07	2,836	374,441	-0.0336905	0.136837	0.805521
2	rs11038895	T	C	0.0831163	0.0166742	6.21E-07	2,836	374,441	0.062055	0.0345699	0.072644
3	rs116952958	A	G	0.187105	0.0402503	3.34E-06	2,836	374,441	0.0600646	0.0881039	0.495399
4	rs11758148	C	A	0.180602	0.019131	3.72E-21	2,836	374,441	-0.0090104	0.0408078	0.825247
5	rs117753409	A	C	0.109919	0.022981	1.73E-06	2,836	374,441	0.0759851	0.0487756	0.119269
6	rs11887597	C	T	-0.0607533	0.0132693	4.68E-06	2,836	374,441	0.00575367	0.0268465	0.8303
7	rs11901096	A	G	0.109304	0.0234208	3.06E-06	2,836	374,441	0.0942482	0.0485472	0.0522132
8	rs12498981	G	A	0.0912604	0.0175126	1.88E-07	2,836	374,441	-0.0395692	0.0364112	0.277156
9	rs12633797	G	A	-0.0662484	0.0138164	1.63E-06	2,836	374,441	0.0123194	0.0278649	0.658408
10	rs12693945	G	A	-0.0733765	0.0155595	2.41E-06	2,836	374,441	-0.0258147	0.0313586	0.410389
11	rs13180950	C	T	0.0912518	0.0179306	3.60E-07	2,836	374,441	0.012012	0.0369801	0.745315
12	rs13419672	T	C	0.188114	0.0390007	1.41E-06	2,836	374,441	-0.0609473	0.0816284	0.455278
13	rs139382712	T	C	0.229431	0.0464976	8.05E-07	2,836	374,441	0.0150225	0.100607	0.881301
14	rs142706153	C	T	-0.290994	0.0623674	3.07E-06	2,836	374,441	0.0803543	0.116603	0.490744
15	rs142770866	A	G	-0.122839	0.0250456	9.36E-07	2,836	374,441	-0.114635	0.0492892	0.0200313
16	rs1432019	T	C	0.0688011	0.0147241	2.97E-06	2,836	374,441	0.0333986	0.0299555	0.264876
17	rs144651842	A	G	0.123185	0.0240429	3.00E-07	2,836	374,441	0.0644825	0.0506065	0.202595
18	rs146771131	C	T	0.0684859	0.0141539	1.31E-06	2,836	374,441	0.032855	0.0288599	0.25494
19	rs16903065	A	C	-0.143169	0.0213703	2.09E-11	2,836	374,441	0.0118031	0.0421238	0.779324
20	rs190146605	C	G	-0.188073	0.0407833	4.00E-06	2,836	374,441	0.0310297	0.0814013	0.703059
21	rs2156698	A	G	-0.0609414	0.0133265	4.81E-06	2,836	374,441	0.0294851	0.0270212	0.275192
22	rs2264583	C	T	0.164927	0.0359557	4.50E-06	2,836	374,441	-0.0135644	0.0686418	0.843349
23	rs2304939	G	A	-0.122462	0.0265554	4.00E-06	2,836	374,441	-0.0836604	0.0518835	0.10686
24	rs2493016	T	C	0.092075	0.0185394	6.82E-07	2,836	374,441	-0.0149587	0.0383242	0.696301
25	rs3108155	G	C	-0.0782243	0.0148348	1.34E-07	2,836	374,441	-0.0259401	0.0296687	0.381942
26	rs3115354	A	G	0.239276	0.0509824	2.69E-06	2,836	374,441	-0.0990303	0.109479	0.365697
27	rs33931897	C	G	0.0629934	0.0137412	4.56E-06	2,836	374,441	-0.00783295	0.0279124	0.778997
28	rs35403056	A	G	-0.0684644	0.0148221	3.85E-06	2,836	374,441	0.0149197	0.0298415	0.617099
29	rs35472395	G	A	-0.677263	0.13789	9.03E-07	2,836	374,441	-0.117899	0.22596	0.601832
30	rs35744187	A	G	0.192444	0.0192957	1.99E-23	2,836	374,441	0.0266194	0.040925	0.515405
31	rs3762333	C	A	0.0740304	0.0158928	3.19E-06	2,836	374,441	0.0181987	0.0328342	0.579401
32	rs3793472	A	G	-0.0760219	0.0153309	7.10E-07	2,836	374,441	0.012028	0.0313917	0.701602
33	rs3986358	C	T	-0.061116	0.0133152	4.43E-06	2,836	374,441	0.00151356	0.0270424	0.955366
34	rs4624466	T	C	0.0841543	0.0173561	1.24E-06	2,836	374,441	0.0560967	0.0358958	0.118108
35	rs548877	G	A	0.0964878	0.0170291	1.46E-08	2,836	374,441	0.026365	0.0351223	0.452856

36	rs6058247	T	C	-0.0759427	0.0164082	3.69E-06	2,836	374,441	-0.0550657	0.0329871	0.0950561
37	rs62054623	G	A	-0.117749	0.0218803	7.39E-08	2,836	374,441	-0.0420211	0.0434765	0.333782
38	rs62127764	G	C	-0.064906	0.0134939	1.51E-06	2,836	374,441	1.40E-05	0.0272236	0.999591
39	rs62242119	T	A	0.138435	0.0298171	3.44E-06	2,836	374,441	0.0409295	0.061634	0.506643
40	rs62405652	C	A	-0.505959	0.025491	1.13E-87	2,836	374,441	-0.144695	0.0476417	0.0023882
41	rs66654254	A	G	-0.0680291	0.0134789	4.49E-07	2,836	374,441	0.0172486	0.0272799	0.527202
42	rs6734893	G	A	0.133673	0.0284428	2.61E-06	2,836	374,441	0.121873	0.0560481	0.0296722
43	rs6777997	G	C	0.0919616	0.018047	3.48E-07	2,836	374,441	0.00282995	0.0372171	0.939388
44	rs72660908	G	C	-0.0640081	0.0139839	4.71E-06	2,836	374,441	0.0205268	0.0280659	0.464548
45	rs72782795	G	A	-0.172334	0.0330331	1.82E-07	2,836	374,441	0.0530452	0.0651637	0.415628
46	rs73170759	T	A	0.16943	0.0341321	6.91E-07	2,836	374,441	-0.0664522	0.071348	0.351656
47	rs75800761	C	T	0.1397	0.0302	3.73E-06	2,836	374,441	-0.00993682	0.0628821	0.874439
48	rs7625875	A	C	0.0676451	0.0146608	3.95E-06	2,836	374,441	-0.026775	0.0295377	0.364688
49	rs7731626	A	G	-0.108014	0.0149385	4.81E-13	2,836	374,441	-0.0423567	0.0298255	0.155565
50	rs77899560	G	A	0.177905	0.037422	1.99E-06	2,836	374,441	-0.0322092	0.0805526	0.689265
51	rs78248443	T	C	-0.178636	0.0262275	9.69E-12	2,836	374,441	-0.0519815	0.0514017	0.311883
52	rs78782944	T	C	0.125311	0.0219434	1.13E-08	2,836	374,441	0.0788363	0.0461427	0.0875367
53	rs8002731	C	A	-0.0902776	0.0138528	7.18E-11	2,836	374,441	0.037195	0.0279049	0.182558
54	rs8121509	C	T	0.0674888	0.0133207	4.05E-07	2,836	374,441	0.00477558	0.0270363	0.859794
55	rs8129030	A	T	0.0695223	0.0139712	6.49E-07	2,836	374,441	0.00373282	0.0280877	0.894274
56	rs9921681	T	C	0.0654854	0.0140387	3.09E-06	2,836	374,441	0.0651447	0.0284916	0.0222275

SNP, single nucleotide polymorphism; EA, effect allele; OA, other allele; SE, standard error; RA, rheumatoid arthritis; GD, Graves disease.

Supplementary Table S3 Information of identified SNPs in exposure (AIT) and outcome (RA)

				Exposure (AIT)			Outcome (RA)				
	SNP	EA	OA	β	SE	*p* value	Case	Control	β	SE	*p* value
1	rs1025256	G	A	0.314269	0.0651234	1.39E-06	3,049	279,760	-0.0167123	0.0143944	0.24563
2	rs10865180	A	G	0.329956	0.0798891	3.62E-05	3,049	279,760	0.00522826	0.0182063	0.773984
3	rs11061760	C	T	0.30745	0.0751239	4.27E-05	3,049	279,760	-0.0056936	0.0169768	0.737342
4	rs11192013	C	T	-0.291002	0.0697478	3.02E-05	3,049	279,760	0.0312432	0.0142406	0.0282384
5	rs111974140	C	T	-0.277266	0.0655465	2.34E-05	3,049	279,760	-0.0205306	0.0136107	0.131448
6	rs112241357	A	G	0.574445	0.138244	3.25E-05	3,049	279,760	0.0704721	0.0360531	0.0506209
7	rs112331534	C	T	0.633624	0.141005	7.00E-06	3,049	279,760	-0.0401532	0.0361761	0.267025
8	rs114644112	G	A	0.753603	0.177922	2.28E-05	3,049	279,760	0.00341053	0.0472904	0.942507
9	rs115513802	C	T	0.588563	0.144354	4.56E-05	3,049	279,760	0.0116949	0.0372309	0.753432
10	rs11706033	T	C	-0.388728	0.0918152	2.30E-05	3,049	279,760	0.00531104	0.0178615	0.766203
11	rs117206250	T	A	1.14957	0.274268	2.77E-05	3,049	279,760	0.0219955	0.0860206	0.798182
12	rs117944015	T	C	1.44667	0.337467	1.81E-05	3,049	279,760	-0.0222519	0.124445	0.858087
13	rs118162083	G	A	1.18895	0.287166	3.47E-05	3,049	279,760	0.0984916	0.0885095	0.265803
14	rs11837712	C	G	0.795142	0.179719	9.67E-06	3,049	279,760	0.0502359	0.048197	0.297271
15	rs12127370	G	A	0.472716	0.108538	1.33E-05	3,049	279,760	0.0433498	0.0259145	0.094367
16	rs12589558	C	T	0.319573	0.0745854	1.83E-05	3,049	279,760	0.0240781	0.0166973	0.149294
17	rs138602074	T	C	-0.880325	0.212512	3.44E-05	3,049	279,760	0.0249791	0.0352486	0.47854
18	rs143412024	A	G	0.87	0.206271	2.47E-05	3,049	279,760	-0.100849	0.0571137	0.0774373
19	rs144513952	A	G	0.667098	0.158488	2.56E-05	3,049	279,760	-0.0410803	0.0408999	0.315181
20	rs145519624	C	A	0.613155	0.1464	2.81E-05	3,049	279,760	0.0352058	0.0377924	0.351566
21	rs149021369	T	C	-1.06865	0.256528	3.10E-05	3,049	279,760	-0.0243471	0.0388293	0.53064
22	rs150596578	T	C	0.884001	0.195784	6.33E-06	3,049	279,760	-0.000988988	0.0545772	0.985542
23	rs150879737	T	C	1.15009	0.264898	1.41E-05	3,049	279,760	-0.0105944	0.0870731	0.903159
24	rs1604966	C	G	0.342512	0.0663884	2.48E-07	3,049	279,760	-0.0152911	0.0148295	0.302483
25	rs16967767	T	C	1.19627	0.29416	4.77E-05	3,049	279,760	-0.241764	0.0969019	0.0125982
26	rs1800009	C	T	0.272537	0.0637515	1.91E-05	3,049	279,760	0.015828	0.013804	0.251537
27	rs214825	C	T	-0.439808	0.105748	3.20E-05	3,049	279,760	0.0501232	0.0249025	0.0441377
28	rs2707275	T	C	0.270325	0.0626247	1.58E-05	3,049	279,760	0.000769742	0.0133708	0.954092
29	rs35173692	A	T	-1.06458	0.257188	3.48E-05	3,049	279,760	0.00182582	0.0414208	0.964841
30	rs35529104	T	C	1.07077	0.261141	4.13E-05	3,049	279,760	0.00852062	0.0823324	0.917574
31	rs4947750	C	T	0.667293	0.160791	3.32E-05	3,049	279,760	0.00955211	0.0409847	0.815711
32	rs532558	C	T	-0.359692	0.0816087	1.05E-05	3,049	279,760	-0.0140401	0.0188347	0.456004
33	rs55906846	G	C	0.509594	0.105867	1.48E-06	3,049	279,760	-0.0243706	0.0258439	0.345685
34	rs56224189	T	G	0.632792	0.150782	2.71E-05	3,049	279,760	-0.0122382	0.0385877	0.751128
35	rs61870853	C	A	1.08581	0.267453	4.91E-05	3,049	279,760	0.0209167	0.0823362	0.799465

36	rs62075181	T	C	0.442435	0.107706	3.99E-05	3,049	279,760	0.0160622	0.0256084	0.530512
37	rs62408211	T	A	0.297321	0.0686763	1.50E-05	3,049	279,760	0.0203125	0.0151726	0.180648
38	rs6785758	A	G	0.364884	0.0810545	6.74E-06	3,049	279,760	-0.00321721	0.0189029	0.864856
39	rs7242091	G	A	-0.27487	0.0649469	2.31E-05	3,049	279,760	-0.00342992	0.0141433	0.808384
40	rs72682840	T	C	-0.626315	0.151106	3.40E-05	3,049	279,760	0.012438	0.0268257	0.642892
41	rs73237426	T	A	0.462271	0.106278	1.36E-05	3,049	279,760	0.0138406	0.0256504	0.589481
42	rs73284905	A	G	0.30827	0.0706825	1.29E-05	3,049	279,760	-0.000204171	0.0157512	0.989658
43	rs76196487	G	A	0.848037	0.200174	2.27E-05	3,049	279,760	-0.0479913	0.0575272	0.404148
44	rs7703397	C	A	0.311465	0.0700069	8.62E-06	3,049	279,760	-0.00409953	0.0142557	0.773676
45	rs77880724	T	A	0.39086	0.0925807	2.42E-05	3,049	279,760	-0.0142361	0.0217491	0.51275
46	rs7823034	A	G	-0.362976	0.0798965	5.54E-06	3,049	279,760	-0.00325071	0.0182601	0.858705
47	rs79339719	A	C	0.606856	0.144127	2.55E-05	3,049	279,760	-0.0773741	0.0360432	0.0318171
48	rs80101832	G	A	-0.297448	0.0713671	3.07E-05	3,049	279,760	0.023416	0.0144735	0.105695
49	rs8088539	A	G	-0.391909	0.0846817	3.69E-06	3,049	279,760	0.00423229	0.019716	0.83003
50	rs8096446	C	T	-0.379343	0.091908	3.67E-05	3,049	279,760	0.00392194	0.0179787	0.827317
51	rs9653729	C	T	0.282481	0.06808	3.34E-05	3,049	279,760	-0.00404555	0.0150408	0.787951

SNP, single nucleotide polymorphism; EA, effect allele; OA, other allele; SE, standard error; RA, rheumatoid arthritis; AIT, autoimmune thyroiditis.

Supplementary Table S4 Information of identified SNPs in exposure (GD) and outcome (RA)

				Exposure (GD)			Outcome (RA)				
	SNP	EA	OA	β	SE	*p* value	Case	Control	β	SE	*p* value
1	rs10199135	G	A	-0.193163	0.039828	1.24E-06	3,049	279,760	-0.0417745	0.0188349	0.0265601
2	rs1051137	T	A	0.11784	0.0273126	1.60E-05	3,049	279,760	0.0122002	0.0136347	0.370901
3	rs10873596	C	T	-0.15299	0.0374178	4.34E-05	3,049	279,760	0.00940657	0.0193324	0.626562
4	rs10896024	G	A	0.111854	0.0275125	4.79E-05	3,049	279,760	0.00967072	0.0135787	0.476342
5	rs1114610	G	A	0.120057	0.029325	4.24E-05	3,049	279,760	0.0139601	0.0148184	0.346151
6	rs113430288	C	T	-0.224978	0.0521583	1.61E-05	3,049	279,760	-0.0012064	0.0247713	0.961157
7	rs115986533	A	T	-0.585482	0.123459	2.11E-06	3,049	279,760	0.0296237	0.0523156	0.571225
8	rs11709131	A	G	0.208484	0.048976	2.07E-05	3,049	279,760	-0.00859325	0.0257715	0.738802
9	rs117366411	T	C	0.94392	0.206169	4.69E-06	3,049	279,760	-0.0842776	0.141154	0.550465
10	rs117537112	A	C	0.35962	0.0842698	1.98E-05	3,049	279,760	0.0489705	0.0464555	0.29182
11	rs11765073	A	C	0.530117	0.119889	9.79E-06	3,049	279,760	-0.0569982	0.070841	0.421054
12	rs12550656	A	G	-0.376446	0.0837497	6.96E-06	3,049	279,760	0.0702941	0.0364136	0.0535525
13	rs12659208	T	C	0.251223	0.0578559	1.41E-05	3,049	279,760	-0.0106259	0.0316058	0.736719
14	rs1290752	G	C	-0.176716	0.0409847	1.62E-05	3,049	279,760	0.0276801	0.0192436	0.150319
15	rs13003541	G	A	0.557909	0.107706	2.22E-07	3,049	279,760	0.105124	0.063944	0.100175
16	rs13213832	G	A	0.139465	0.0331985	2.66E-05	3,049	279,760	-0.00834209	0.0168676	0.620908
17	rs137939567	G	C	0.274528	0.0672854	4.50E-05	3,049	279,760	0.0097545	0.0361261	0.787151
18	rs1383132	G	A	0.120122	0.0282373	2.10E-05	3,049	279,760	-0.0225135	0.0141891	0.112587
19	rs139090997	A	G	-0.568778	0.130248	1.26E-05	3,049	279,760	0.0241877	0.0538459	0.653285
20	rs139991771	T	C	-0.465505	0.10442	8.27E-06	3,049	279,760	0.0157305	0.0445406	0.723959
21	rs140430242	T	C	0.39962	0.0786813	3.79E-07	3,049	279,760	-0.016123	0.0439953	0.714014
22	rs141569578	T	A	-0.415974	0.0937475	9.11E-06	3,049	279,760	-0.0743171	0.0409111	0.0692867
23	rs142112070	A	C	0.247293	0.0581643	2.12E-05	3,049	279,760	0.0653551	0.03095	0.0347176
24	rs1426841	A	G	-0.122169	0.0271906	7.02E-06	3,049	279,760	-0.00121608	0.0134061	0.927722
25	rs143159065	T	C	-0.278055	0.067732	4.04E-05	3,049	279,760	-0.0644442	0.0308619	0.0367849
26	rs145008938	T	A	-0.541837	0.0908629	2.47E-09	3,049	279,760	-0.039921	0.0383058	0.297335
27	rs145969285	T	G	-0.334069	0.0818113	4.44E-05	3,049	279,760	0.0141146	0.0364791	0.698813
28	rs147729901	A	G	0.322812	0.0729792	9.72E-06	3,049	279,760	-0.00822446	0.0399927	0.837065
29	rs148171793	C	T	-0.41591	0.0939815	9.62E-06	3,049	279,760	0.0166876	0.0407497	0.682161
30	rs1509973	G	A	0.154059	0.0348008	9.56E-06	3,049	279,760	0.0171897	0.0178705	0.336097
31	rs151069963	C	T	-0.341625	0.078267	1.27E-05	3,049	279,760	-0.0502796	0.0348267	0.14882
32	rs1532480	T	G	-0.112457	0.0275815	4.56E-05	3,049	279,760	-0.00478415	0.0138521	0.729814
33	rs16844050	A	G	-0.16809	0.0384238	1.22E-05	3,049	279,760	-0.000544382	0.0183602	0.976346
34	rs16963351	A	C	0.231456	0.0541191	1.90E-05	3,049	279,760	0.0479823	0.0290362	0.0984328
35	rs17066480	C	A	-0.172815	0.0416537	3.34E-05	3,049	279,760	-0.0462363	0.0198057	0.01957

36	rs17334305	T	C	-0.461783	0.103452	8.05E-06	3,049	279,760	-0.0177608	0.0449426	0.692703
37	rs17513810	C	A	-0.442813	0.0854443	2.19E-07	3,049	279,760	0.0476001	0.0368988	0.197045
38	rs17634990	T	C	-0.183937	0.0445133	3.59E-05	3,049	279,760	0.0205592	0.0212148	0.332498
39	rs181347739	C	T	0.250023	0.0614174	4.68E-05	3,049	279,760	0.00774751	0.0331815	0.815382
40	rs1861628	G	A	-0.115687	0.027224	2.14E-05	3,049	279,760	-0.0021862	0.0136299	0.872568
41	rs1884932	G	A	-0.158033	0.0355579	8.81E-06	3,049	279,760	-0.0226188	0.0182299	0.214697
42	rs193170273	T	C	0.484329	0.109376	9.51E-06	3,049	279,760	-0.0364	0.064148	0.570416
43	rs1985790	A	G	-0.197121	0.027513	7.80E-13	3,049	279,760	0.0209067	0.0136003	0.12424
44	rs1997397	C	T	0.127441	0.0276719	4.12E-06	3,049	279,760	-0.00632967	0.013928	0.6495
45	rs2048496	G	A	-0.179266	0.0388097	3.85E-06	3,049	279,760	-0.0244474	0.0184923	0.186157
46	rs2160215	C	T	0.320977	0.0277957	7.58E-31	3,049	279,760	0.0256434	0.0145314	0.0776176
47	rs2173243	G	A	-0.133584	0.0318398	2.72E-05	3,049	279,760	0.0101614	0.0162433	0.531593
48	rs238947	C	T	0.207012	0.0500338	3.51E-05	3,049	279,760	-0.000153181	0.0261069	0.995318
49	rs242026	T	C	-0.146918	0.0334842	1.15E-05	3,049	279,760	-0.0154308	0.0161499	0.339338
50	rs2466074	T	C	-0.127271	0.0273456	3.25E-06	3,049	279,760	-0.00747414	0.0134811	0.579294
51	rs2514466	C	T	0.111171	0.0274065	4.98E-05	3,049	279,760	0.0056273	0.0137294	0.6819
52	rs251453	G	C	-0.120883	0.0282312	1.85E-05	3,049	279,760	0.0154522	0.0138511	0.264596
53	rs2792043	C	A	0.14022	0.0265871	1.33E-07	3,049	279,760	-0.000127507	0.0132047	0.992296
54	rs28989481	G	C	-0.667771	0.164157	4.74E-05	3,049	279,760	-0.0329746	0.0671361	0.623313
55	rs3136155	T	C	-0.130823	0.0304917	1.78E-05	3,049	279,760	-0.0409519	0.0149391	0.0061204
56	rs34953613	C	T	-0.255387	0.0552325	3.77E-06	3,049	279,760	-0.0177113	0.0256039	0.489099
57	rs350066	A	G	-0.112596	0.0272953	3.71E-05	3,049	279,760	-0.0322334	0.0134419	0.0164862
58	rs35811474	T	C	-0.191966	0.0397939	1.41E-06	3,049	279,760	-0.0192861	0.0188394	0.305974
59	rs373472462	G	A	-0.306391	0.0714236	1.79E-05	3,049	279,760	0.0355864	0.0322302	0.269536
60	rs3942413	C	T	-0.167258	0.041228	4.97E-05	3,049	279,760	-0.00434681	0.021389	0.838958
61	rs41305451	C	T	0.171543	0.0383681	7.79E-06	3,049	279,760	0.00051875	0.0197343	0.979029
62	rs4338740	C	T	0.148498	0.0282315	1.44E-07	3,049	279,760	-0.00976143	0.0142795	0.494229
63	rs45561635	C	A	-0.296687	0.0721374	3.91E-05	3,049	279,760	0.015262	0.0329585	0.643317
64	rs4666559	A	C	-0.161558	0.0378747	1.99E-05	3,049	279,760	0.0163499	0.0183843	0.37382
65	rs5014866	A	G	-0.127302	0.0305771	3.14E-05	3,049	279,760	0.00317764	0.0149408	0.831575
66	rs55800653	C	G	-0.19003	0.0437063	1.37E-05	3,049	279,760	-0.0247982	0.0207495	0.232039
67	rs55859894	A	G	0.255238	0.0602687	2.29E-05	3,049	279,760	0.0493861	0.0326452	0.130327
68	rs55987922	A	T	-0.154611	0.0370135	2.95E-05	3,049	279,760	-0.00401472	0.0190725	0.833279
69	rs56738967	C	G	0.20663	0.0281851	2.28E-13	3,049	279,760	0.00860263	0.0143965	0.550141
70	rs60946162	T	C	-0.161299	0.0273657	3.76E-09	3,049	279,760	-0.0247088	0.0134576	0.0663499
71	rs61734579	C	A	-0.532864	0.0820605	8.38E-11	3,049	279,760	-0.00251012	0.0338245	0.940843
72	rs62125158	A	G	-0.120034	0.027955	1.76E-05	3,049	279,760	-0.031821	0.0137326	0.0204932
73	rs62270889	A	G	-0.12317	0.0294293	2.85E-05	3,049	279,760	-0.0258366	0.0144101	0.072981
74	rs62281990	C	T	-0.362072	0.0865306	2.86E-05	3,049	279,760	0.0384254	0.038324	0.316033

75	rs630659	A	G	-0.121709	0.029383	3.44E-05	3,049	279,760	-0.0130377	0.0148304	0.379335
76	rs6476674	C	T	-0.496473	0.120231	3.64E-05	3,049	279,760	0.00792097	0.0526344	0.880378
77	rs6485123	A	C	0.203801	0.048258	2.41E-05	3,049	279,760	0.00200118	0.0226983	0.929746
78	rs6503208	C	T	-0.137621	0.0312914	1.09E-05	3,049	279,760	8.06E-05	0.0152326	0.995778
79	rs6540172	C	T	0.114437	0.0278016	3.85E-05	3,049	279,760	0.0227065	0.0136345	0.0958385
80	rs6699121	C	G	0.230948	0.0551087	2.78E-05	3,049	279,760	-0.028471	0.0293381	0.331826
81	rs6943937	G	A	-0.133291	0.0283635	2.61E-06	3,049	279,760	-0.00115825	0.0143523	0.93568
82	rs71604684	C	A	0.168629	0.0407786	3.55E-05	3,049	279,760	0.00247738	0.0210007	0.906094
83	rs72735691	C	T	0.529686	0.128316	3.66E-05	3,049	279,760	0.0649143	0.0781831	0.406378
84	rs72958822	T	C	0.598682	0.138143	1.47E-05	3,049	279,760	0.0527688	0.0833715	0.526776
85	rs73002754	C	G	-0.240385	0.0580621	3.47E-05	3,049	279,760	0.0153807	0.0271034	0.570388
86	rs73044684	C	G	0.137509	0.0326136	2.48E-05	3,049	279,760	0.00225652	0.0166325	0.892083
87	rs74276436	C	T	-0.367522	0.0903904	4.78E-05	3,049	279,760	-0.0929921	0.0399107	0.0198061
88	rs74582032	A	G	-0.391905	0.0859904	5.18E-06	3,049	279,760	0.0656927	0.0378617	0.0827275
89	rs74957012	A	G	-0.430402	0.0949801	5.86E-06	3,049	279,760	0.0570539	0.04073	0.161279
90	rs75306313	C	G	0.156865	0.0373331	2.65E-05	3,049	279,760	0.00407115	0.0191914	0.832003
91	rs76812815	A	C	-0.341543	0.0841037	4.89E-05	3,049	279,760	-0.0403453	0.0376939	0.284465
92	rs77186266	G	T	-0.174313	0.0367719	2.13E-06	3,049	279,760	0.00545084	0.0175478	0.756084
93	rs78121205	T	G	0.175361	0.0423445	3.45E-05	3,049	279,760	-0.0175448	0.0219604	0.424333
94	rs80044667	C	G	0.4495	0.101253	9.02E-06	3,049	279,760	0.0550047	0.0582836	0.345301
95	rs80217563	T	C	0.232794	0.0501215	3.41E-06	3,049	279,760	-0.000707702	0.0263157	0.978545
96	rs8125909	C	A	0.222709	0.0546057	4.53E-05	3,049	279,760	0.00990498	0.0292204	0.734629
97	rs9833157	A	G	0.118236	0.0282149	2.78E-05	3,049	279,760	-0.00571178	0.013892	0.680957
98	rs10199135	G	A	-0.193163	0.039828	1.24E-06	3,049	279,760	-0.0417745	0.0188349	0.0265601

SNP, single nucleotide polymorphism; EA, effect allele; OA, other allele; SE, standard error; RA, rheumatoid arthritis; GD, Graves disease.
