# Supplementary material for: Interference between rheumatoid arthritis and autoimmune thyroid diseases: A bidirectional Mendelian randomization
Source: Medicine (Baltimore). 2025 Apr 18;104(16):e42188. doi: 10.1097/MD.0000000000042188 (PMC12014076; doi:10.1097/MD.0000000000042188)
Supplement: Supplementary file 2 [file medi-104-e42188-s002.docx]

**Supplementary Table S5** MR-Egger intercept analysis of horizontal pleiotropy

| **Exposure** | **Outcome** | **Egger_intercept** | **SE** | ***p* value** |
| --- | --- | --- | --- | --- |
| RA | AIT | -0.025780159 | 0.021191159 | 0.228538588 |
| RA | GD | -0.008534427 | 0.00950227 | 0.373094933 |
| AIT | RA | 0.003687774 | 0.008122596 | 0.651823297 |
| GD | RA | 0.007203037 | 0.004981875 | 0.15151066 |

RA, rheumatoid arthritis; AIT, autoimmune thyroiditis; GD, Graves disease.
