# Supplementary material for: Interference between rheumatoid arthritis and autoimmune thyroid diseases: A bidirectional Mendelian randomization
Source: Medicine (Baltimore). 2025 Apr 18;104(16):e42188. doi: 10.1097/MD.0000000000042188 (PMC12014076; doi:10.1097/MD.0000000000042188)
Supplement: Supplementary file 3 [file medi-104-e42188-s003.docx]

**Supplementary Table S6 Cochrane Q test of heterogeneity analysis**

| **Exposure** | **Outcome** | **Method** | **Q** | **Q_df** | **Q_*p* val** |
| --- | --- | --- | --- | --- | --- |
| RA | AIT | MR Egger | 43.006497 | 60 | 0.952098654 |
| RA | AIT | Inverse variance weighted | 44.4864971 | 61 | 0.94458273 |
| RA | GD | MR Egger | 54.12847801 | 54 | 0.469492131 |
| RA | GD | Inverse variance weighted | 54.93706367 | 55 | 0.477024919 |
| AIT | RA | MR Egger | 51.92860496 | 49 | 0.360464358 |
| AIT | RA | Inverse variance weighted | 52.14705363 | 50 | 0.390428599 |
| GD | RA | MR Egger | 106.4321495 | 95 | 0.198724606 |
| GD | RA | Inverse variance weighted | 108.7741929 | 96 | 0.175787314 |

RA, rheumatoid arthritis; AIT, autoimmune thyroiditis; GD, Graves disease.
